# Notes and News

**Published:** 1900-11-24

**Authors:** 


### NOTES AND NEWS.

MR. AND MRS. BARTRAM have given one thousand guineas to the Sunderland Infirmary to endow a bed for the use of female patients. The donation is made in testimony of the excellence of the institution.

THE Chinese laundry hands, of the employment of which we pointed out the dangers some little time past, have already got into trouble. They have been unable to get their wages for some weeks past owing, the employers state, to the withholding of the money by the person to whom it was paid as their deputy.

AT the annual meeting of the Royal College of Surgeons held last week Sir William MacCormac, the President entered into the question in dispute between the Royal College and the General Medical Council as to the requirements of the latter body regarding the commencement of medical study, and stated that they considered the regulation which had been made by the General Medical Council to be an encroachment on their rights. So the deadlock continues. Various resolutions as to "Reform" and other matters were carried as usual, without apparently in any way disturbing the equanimity of the Council.

THE Metropolitan Asylums Board are about to apply to the Local Government Board to sanction a loan of £284,312 for the purpose of establishing a new hospital at Carshalton, to be called the Southern Hospital. This great hospital will contain 800 beds, the peculiarity in its construction being the adoption of the cottage or isolated block system in order to ensure more complete classification and separation of the patients suffering from different diseases than has hitherto been possible. The committee reported that they had carefully gone into the question of cost, and that in the light of their representation to their professional advisers it had been found possible to reduce the estimate for the construction, fitting up, and furnishing of the hospital by £32,100, namely, from £316,400 to £284,300, the cost per bed being thereby reduced from £395 to about £355. Mr. J. Brown, the chairman of the committee, stated that many costly items have been omitted without in any way impairing the efficiency of the hospital, and Mr. Crooks congratulated the Board on the moral courage it had at last displayed in criticising plans sent in by architects and estimates founded upon them.

THE Hospital for Diseases of the Chest, City Road, will hold a festival dinner in May next to aid in securing a nurses' home for the hospital. Sir T. Andros de la Rue will preside.

THE epidemic of typhoid at Plymouth shows no abatement as yet. There are upwards of fifty cases under treatment. Nearly all the patients are children. The infection, which spread from the suburb of Mutley, is traced to a supply of milk coming from an infected source.

A NEW Maternity Hospital has been opened at Aberdeen. The building is not a new one, having been purchased and converted to its present use for the sum of £4,000. The work of the management has been seriously handicapped by unsuitable premises in the past, and the present site and the arrangements of the hospital are pronounced satisfactory, and in every way an improvement.

ON the last day of the existence of the Health Committee of the late Vestry of St. George-the-Martyr, Southwark, as a separate sanitary authority, Dr. Waldo, medical officer of health, presented a report on the recent outbreak of enteric fever in the parish. It had been impossible to discover the origin of the outbreak, there not being sufficient evidence to connect it with either milk, water, ice cream, water-ices, shell-fish, swimming baths, or other polluted condition of environment. Dr. Waldo said that the safety of the parish would be increased if arrangements were made for placing Widal's test at the free disposal of the medical practitioners in the district, and he added that there were in the neighbourhood various excellent laboratories which would be available for the purpose.

A SPECIAL COMMITTEE has been considering the report made upon the present condition of the Tunbridge Wells Hospital by Mr. Michelli, Secretary of the Seamen's Hospital, Greenwich. Mr. Michelli advocated the building of a new hospital. The Committee recommend that this suggestion should be followed, the present site being adhered to. Mr. Percy Adams, the architect, has advised that the present hospital be utilised for administration and domestic purposes, and as a nurses' home. The new buildings to consist of three pavilions, to contain seventy-six patients, connected by corridors with the present building; a new out-patients' department, kitchen, laundry, and boiler house, the estimated cost being £26,950. The Committee have fully endorsed Mr. Percy Adams's proposed scheme, and have advised the Governors to adopt it.

THE half-yearly report issued by Dr. Collingridge, the medical officer of health for the Port of London, shows how much good work is being done by the inspection of shipping down the river. Among the cases of infectious disease dealt with were two cases of plague. A great and important work is being done in the inspection of imported food, large quantities of frozen beef, lamb, and mutton having been seized and condemned as unfit for food, together with 3,283 crates of rabbits, 3,880 loose rabbits, 9,431 cases of green fruit, 1,015 tins of preserved fruit, and 21,418 packages of sundries. It is well that Londoners should be reminded now and again of the large amount of unobtrusive sanitary work which goes on down the river for their protection—work often done in the worst of weathers and in the midst of very trying surroundings.

